# Transformed Lymphoma Is Associated with a Favorable Response to CAR-T-Cell Treatment in DLBCL Patients

**DOI:** 10.3390/cancers13236073

**Published:** 2021-12-02

**Authors:** Anna Nydegger, Urban Novak, Marie-Noëlle Kronig, Myriam Legros, Sacha Zeerleder, Yara Banz, Ulrike Bacher, Thomas Pabst

**Affiliations:** 1 Department of Medical Oncology, Inselspital, Bern University Hospital, 3010 Bern, Switzerland; anna.nydegger@students.unibe.ch (A.N.); urban.novak@insel.ch (U.N.); marie-noelle.kronig@insel.ch (M.-N.K.); 2 Center of Laboratory Medicine (ZLM), Inselspital, Bern University Hospital, 3010 Bern, Switzerland; myriam.legros@insel.ch; 3 Department of Hematology, Inselspital, Bern University Hospital, 3010 Bern, Switzerland; sacha.zeerleder@insel.ch (S.Z.); veraulrike.bacher@insel.ch (U.B.); 4 Institute of Pathology, Inselspital, University of Bern, 3008 Bern, Switzerland; yara.banz@pathology.unibe.ch

**Keywords:** CAR-T-cell therapy, diffuse large B-cell lymphoma (DLBCL), secondary DLBCL, prognosis, relapse

## Abstract

**Simple Summary:**

The clinical features predicting favorable outcomes after CAR-T-cell treatment are a matter of ongoing debate. This study aimed to evaluate the potential importance of lymphoma subtypes regarding prognostic significance, mainly to compare transformed versus de novo DLBCL. We found that patients with transformed/secondary lymphoma have a decisively more favorable course after CAR-T-cell therapy than patients with de novo lymphoma.

**Abstract:**

(1) Background: CAR-T-cell therapy is a novel therapeutic option for patients with relapsed/refractory diffuse large B-cell lymphoma (*r*/*r* DLBCL). The parameters that predict a favorable outcome after CAR-T-cell treatment are a matter of ongoing exploration. (2) Methods: We analyzed 36 consecutive patients with *r*/*r* DLBCL receiving tisagenlecleucel or axicabtagene ciloleucel at a single academic institution. We hypothesized that lymphoma subtypes (transformed versus de novo DLBCL) are of prognostic importance. We also assessed age, previous treatment, bridging therapy, remission status at the time of CAR-T treatment and at six months, LDH, the occurrence of CRS or ICANS, and CAR-T-DNA ddPCR kinetics for their prognostic impact. (3) Results: CRS was observed in 24 (67%) patients, and ICANS was observed in 14 (39%) patients. CR was achieved in 20 (56%) patients. Achievement of CR within six months after CAR-T was associated with better PFS (*p* < 0.0001) and OS (*p* < 0.0001). Remarkably, transformed (=secondary) lymphoma was associated with a better outcome than de novo disease for PFS (*p* = 0.0093) and OS (*p* = 0.0209), and the CR rate was 78% versus 33% (*p* = 0.0176). Mortality in patients with transformed DLBCL was 23% compared with 56% in de novo patients (*p* = 0.0209). (4) Conclusion: The presence of transformed DLBCL seems to be associated with a more favorable course after CAR-T treatment than that observed in the de novo DLBCL patients.

## 1. Introduction

Currently, chimeric antigen receptor T-cell therapy (CAR-T) is in the process of being introduced in an increasing number of centers for patients with refractory or relapsed aggressive B-cell lymphoid malignancies. So far, approved CAR-T-cell products for such patients rely on autologous genetically engineered T-lymphocytes, which express a chimeric antigen receptor (CAR) against the pan-B-cell CD19 antigen [1,2,3,4,5,6,7]. The basic anatomy of a CAR structure consists of an antigen-recognition domain, usually a single-chain variable fragment derived from a monoclonal antibody targeting the selected antigen (i.e., CD19); a hinge (usually derived from CD8 or Ig4 molecules) that links the recognition site to the transmembrane domain, which bridges the membrane; and finally, the intracellular domain that typically contains the CD3z chain critical for T-cell receptor signaling [8]. T-cell engineering started in 1990 with the development of the first generation of CAR-T cells in 1993; however, their clinical effectiveness was reduced due to their short persistence. After the introduction of a co-stimulatory domain in 1998, second-generation CARs were introduced in 2003. They were built to target CD19, and their discovery led to the first success in the treatment of aggressive lymphatic malignancies in 2011. The CD19 antigen is a transmembrane glycoprotein involved in regulating B-lymphocyte activation through humoral signaling [8]. CD19 is an ideal target that is ubiquitously expressed in B-lymphocytes, and its expression is maintained during lymphoid B-cell maturation and, importantly, neoplastic transformation [9].

Based on the JULIET trial [2,6,10,11], tisagenlecleucel (tisa-cel) was approved by the European Medicines Agency (EMA) in 2018 for refractory/relapsed (*r*/*r*) diffuse large B-cell lymphoma (DLBCL) after at least two previous treatment lines as well as for *r*/*r* B-cell acute lymphoblastic leukemia (B-ALL) in children and young adults up to 25 years of age with failure or relapse after two previous therapies or following allogeneic hematopoietic stem-cell transplantation (HSCT). Tisa-cel is a second-generation CAR-T utilizing 4-1BB as a co-stimulatory domain, which was developed by the investigators of the University of Pennsylvania in collaboration with Novartis [2,6]. Axicabtagene ciloleucel (axi-cel) is a second CAR-T-cell product, which the EMA authorized in 2018 for *r*/*r* DLBCL and primary mediastinal large B-cell lymphoma (PMBCL) after at least two lines of therapy [3]. It is a second-generation CAR-T utilizing a CD28 co-stimulatory domain. It was initially developed by the investigators of the National Cancer Institute (NCI). Currently, the construct is being developed by Kite Pharma, Gilead Sciences, and Daiichi Sankyo [3]. Lisocabtagene maraleucel (liso-cel) is currently available for relapsed/refractory DLBCL, PMBCL, follicular lymphoma Grade 3B, and mantle cell lymphoma (MCL) and is in the process of obtaining market authorization [4].

Despite the rapidly increasing number of clinical reports, uncertainty remains regarding the factors that impact clinical outcomes after CAR-T-cell treatment [4,5,6,12,13,14,15]. In particular, it remains unclear whether distinct lymphoma subtypes may differ in their response to CAR-T-cell treatment [1,6,7,16,17,18]. For example, a recent report suggested that patients with a history of follicular lymphoma had a better progression-free course than patients with DLBCL [16]. In addition, the TRANSCEND study reported similar findings [4]. Consequently, we investigated clinical and laboratory parameters in a cohort of subsequent patients with lymphoid malignancies for their prognostic significance, focusing on elucidating the impact of transformed (or secondary) versus de novo (or primary) DLBCL histology.

## 2. Materials and Methods

We conducted a prospective single-center study at the University Hospital Inselspital, Bern, Switzerland. The cohort comprised all consecutive patients who underwent commercial CAR-T-cell therapy between January 2019 and June 2020. All participants gave written informed consent for the usage of personal data for research purposes, and a decision by the local ethics committee approved this study.

All patients were followed throughout the CAR-T-cell process until at least six months after the CAR-T-cell infusion. In addition, clinical and laboratory data related to the underlying disease, the CAR-T-cell procedure, clinical complications, and survival endpoints were collected. Parameters investigated for their potential prognostic significance were: patient age, transformed versus de novo disease, previous treatment lines, the need for bridging therapy before CAR-T-cell infusion, remission status at the time of CAR-T treatment, levels of serum ferritin (17), lactate dehydrogenase (LDH) before lymphodepleting therapy, use of Kymriah^®^ versus Yescarta^®^ CAR-T-cell products, the manifestation of a cytokine release syndrome (CRS) and/or an immune effector cell-associated neurotoxicity syndrome (ICANS), peak CAR-T-DNA concentration and kinetics during follow-up by digital droplet polymerase chain reaction (ddPCR) in the peripheral blood (19), and remission status at six months after CAR-T-cell infusion.

The progression-free survival (PFS) and overall survival (OS), by the Kaplan–Meier method, were defined as the time from CAR-T-cell infusion to disease progression, death, or last follow-up, respectively, and compared by a log-rank (Mantel–Cox) test. PFS and OS were censored at the last follow-up on 1 February 2021, which was also used as the data cutoff. The follow-up of CAR-T-DNA concentrations in peripheral blood continued beyond the data cutoff.

For the univariate analyses, we used GraphPad Prism version 8, and we performed univariate analyses for the prognostic parameters listed above. The categorical variables were summarized as frequencies and percentages, and the continuous variables were summarized as medians and ranges.

## 3. Results

### 3.1. Clinical Characteristics of the Patients

The clinical characteristics of the 36 patients are summarized in Table 1. The median age of the patients at the time of CAR-T therapy was 68.8 years. DLBCL patients showed an equal distribution between de novo and transformed DLBCL (de novo DLBCL 50%, 18/36; transformed DLBCL 50%, 18/36). The primary diagnosis in transformed DLBCL patients was follicular lymphoma (FL; *n* = 13), chronic lymphatic leukemia (CLL; *n* = 2)/small lymphocytic lymphoma (SLL; *n* = 1), marginal-zone lymphoma (MZL; *n* = 1), and other lymphoma (*n* = 1). The majority of the patients had previously received two treatment lines (61%, 22/36), with the remaining patients having three or more previous treatment lines (39%, 14/36).

### 3.2. Clinical Status at the Time of CAR-T Infusion and Therapeutic Course

We present the clinical status at the time of CAR-T-cell infusion in Table 2 and the clinical course after the CAR-T therapy in Table 3. At the time of CAR-T-cell treatment, 17 patients had a progressive disease (PD, 47%, 17/36), 8 had a stable disease (SD, 22%, 8/36), 9 were in partial remission (PR, 25%, 9/36), and 2 were in CR (6%, 2/36).

Almost half of the patients (47%, 17/36) received one of the following bridging therapies: R-GEMOX (rituximab, gemcitabine, and oxaliplatin); MATRIx (Mabthera/rituximab, cytarabine, high-dose methotrexate and thiotepa); BR (bendamustine/rituximab) combined with, or without, polatuzumab vedotin, ibrutinib, gemcitabine monotherapy, bendamustine monotherapy, polatuzumab vedotin monotherapy, and rituximab monotherapy. In addition, four patients received radiotherapy as a bridging strategy (alone in one case, combined with chemo-/immunotherapy in three cases).

All patients received lymphodepleting chemotherapy for three days (day −5 to −3) of 300 mg/m^2^ cyclophosphamide and 30 mg/m^2^ fludarabine, with two days of wash-out before the CAR-T-cell infusion (day 0). Twenty-six patients received Kymriah^®^ (Novartis, tisagenlecleucel; 72%), and 10 patients had Yescarta^®^ (Gilead, axicabtagene ciloleucel; 28%).

More than half of the patients experienced CRS of any grade (67%, 24/36; grade 2–4 in 18 patients). ICANS, of any grade, was seen in 14 patients (39%, 14/36; grade 2–4 in 10 patients). The majority of these patients had CRS and/or ICANS of lower grades. CRS of grades 2 and higher was treated with tocilizumab. Short-term admission to the ICU was necessary in one case due to the profound unilateral lung edema and unstable vital parameters. ICANS of grades 2 and higher was treated mainly by steroids. One patient with grade 3 and two patients with grade 4 ICANS had to be admitted to the ICU. Finally, the median hospitalization duration was 21 days, ranging between 18 and 51 days.

### 3.3. Clinical Course and Characteristics of the Patients after CAR-T-Cell Therapy

The best response following CAR-T-cell therapy was CR in 20 patients (56% of the cohort), PR in 7 patients (19%); SD was seen in 6 patients (17%), and PD was seen in 3 patients (8%). At six months after the CAR-T-cell infusion, the response status was CR in 16 patients (44% of the cohort); PR in 5 patients (14%), SD in 1 patient (3%), and PD in 14 patients (39%) (Table 2).

**Figure 1:**
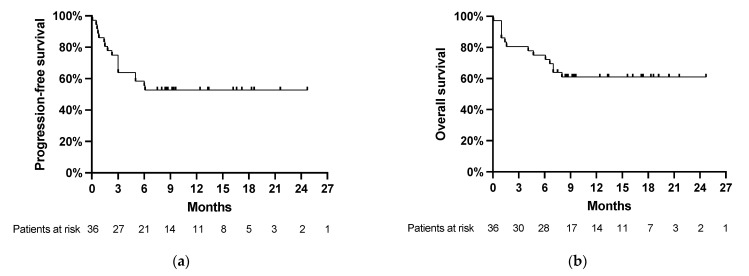
(**a**) Progression-free survival and (**b**) overall survival after CAR-T-cell treatment in the total cohort. Starting time of PFS and OS is on the day of CAR-T infusion (day 0). The last follow-up and data cutoff were on 1 February 2021.

Relapses occurred in 17/36 patients (47%) after a median interval of 70 days (range, 2 to 185 days) after the CAR-T-cell infusion. Death occurred in 14/36 patients (39%) after a median interval of 88 days (range, 2 to 248 days). Thus, most relapses and deaths occurred within the first six months after the CAR-T-cell infusion (Figure 1a,b).

Ten out of the seventeen patients with DLBCL relapses following CAR-T-cell infusion received salvage therapies (28%). Six patients had one line, and four patients had two lines. Treatment included (alone or in combinations) R-MTX-araC, R-BAC, bendamustine, cladribine, lenalidomide, polatuzumab vedotin, daratumumab, rituximab, blinatumomab, obinutuzumab, and glofitamab. In addition, four patients received radiotherapy (in two cases alone and in two cases combined with systemic approaches).

**Figure 2:**
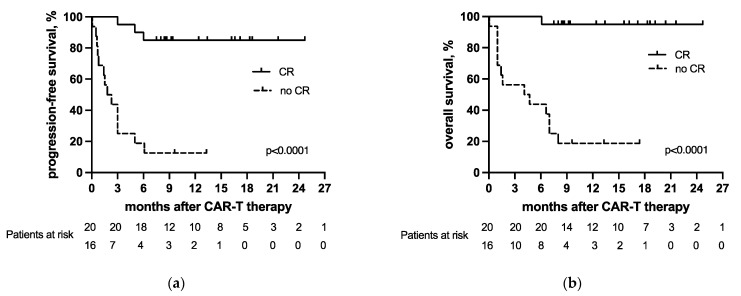
(**a**) Progression-free survival and (**b**) overall survival of the patients who did and did not achieve complete remission as a best response after CAR-T-cell therapy.

Responses at the last follow-up were CR in 53% (19/36) and PR in 5% (2/36). Fifteen patients had PD (42%, 15/36), and 14 patients had died, all due to the underlying lymphoma (39%, 14/36), after a median interval of 88 days (range, 2 to 248 days) since the CAR-T-cell infusion. The patients achieving CR (56%, 20/36) as a best response within the first six months after CAR-T had better progression-free and overall survival than patients without CR (44%, 16/36) (for PFS, *p* < 0.0001; for OS, *p* < 0.0001; Figure 2a,b). We observed no differences in PFS (*p* = 0.1666) or OS (*p* = 0.4444) between patients receiving Kymriah or Yescarta.

The kinetics of the CAR-T-cell-specific DNA in the peripheral blood of the CAR-T-cell recipients were monitored by ddPCR. The median time to reach the peak concentration of the CAR-T-cell-specific DNA in the peripheral blood after the CAR-T-cell infusion was ten days (range, 2 to 29 days). The median peak concentration of CAR-T-cell-specific DNA was 6.506 copies per μg of DNA (range, 54 to 139.656). CAR-T-cell-specific DNA levels were also assessed at 1, 3, 6, and 12 months after the CAR-T-cell infusion. The median CAR-T-cell-specific DNA concentrations were 467 copies per μg of DNA (1st month), 150 copies per μg of DNA (3rd month), 91.5 copies per μg of DNA (6th month), and 97.5 copies per μg of DNA (12th month).

### 3.4. Transformed Lymphoma as a Prognostic Parameter

We investigated the prognostic impact of histopathology: transformed versus de novo DLBCL. Table 4 summarizes the comparison between the two groups. 

**Figure 3:**
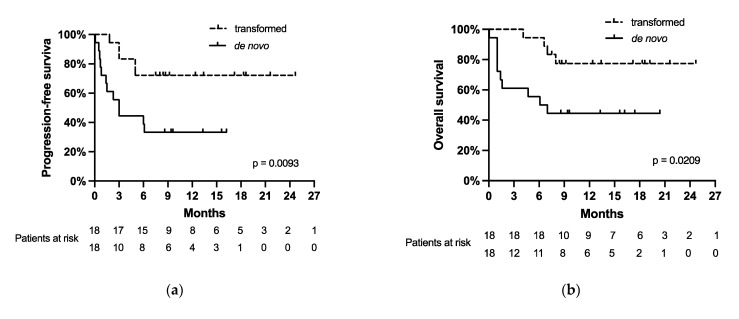

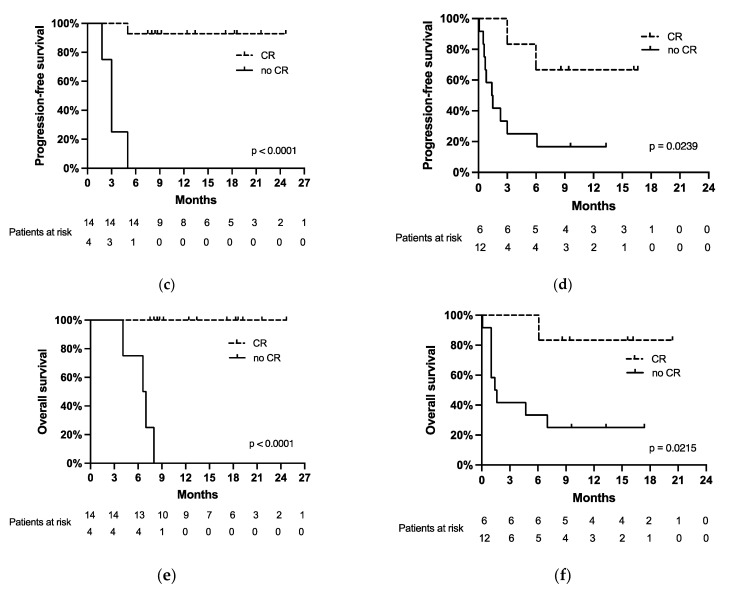
(**a**) Progression-free survival and (**b**) overall survival of the patients with de novo and transformed DLBCL. Progression-free survival of (**c**) patients with transformed lymphoma who reached a complete remission (CR) and who did not; and (**d**) of patients with de novo lymphoma who reached a CR and who did not. Overall survival of (**e**) patients with transformed lymphoma who reached a CR and who did not and (**f**) of patients with de novo lymphoma who reached a CR and who did not.

In the transformed lymphoma group (Figure 3c,e), 14 patients (78%, 14/18) reached CR, and 4 patients (22%, 4/18) did not (median PFS for no CR was 3 months, and median OS for no CR was 6.6 months with *p* < 0.0001 for both PFS and OS).

In contrast, in the de novo DLBCL subgroup (Figure 3d,f), 6 patients (33%; 6/18) achieved CR as a best response, and 12 patients (67%, 12/18) did not (median PFS for no CR was 1.45 months with *p* = 0.0239, median OS for no CR was 1.5 months with *p* = 0.0215). The CR rate was higher in patients with transformed lymphoma compared to those with de novo disease (78% in the transformed group vs. 33% in the de novo group, *p* = 0.0176).

The majority of the patients that relapsed (71%, 12/17—in both subgroups) and died (71%, 10/14—in both subgroups) after CAR-T-cell therapy had de novo DLBCL. In particular, the relapse rate of the patients with de novo lymphoma was 67% (12/18), and the mortality rate was 56% (10/18). Disease progression was the cause of death in all cases. In the transformed lymphoma group, the relapse rate was 28% (5/18), and the mortality rate was 22% (4/18). In addition, we found a significant correlation between having an event (relapse, *p* = 0.0093; or death, *p* = 0.0209) and a diagnosis of de novo DLBCL by univariate analysis (Figure 3a,b).

Survival outcomes, according to the Kaplan–Meier method, were as follows: the median OS was 6.6 months in patients with de novo lymphoma compared to not being reached in transformed lymphoma (*p* = 0.0209), and the median PFS was 3 months in de novo lymphoma versus the median PFS not being reached in transformed lymphoma (*p* = 0.0093). Thus, transformed DLBCL had a significantly lower risk of death of only 23% compared to 56% in those with de novo DLBCL.

In terms of CAR-T-cell therapy-specific complications, no significant differences were observed between the two groups. The rates of patients with CRS (of all grades in 24 patients, in the transformed group 46%, 11/24, and in the de novo group 54%, 13/24) and with ICANS (of all grades in 14 patients, in the transformed group 57%, 8/14, and in the de novo group 43%, 6/14) were similar in both groups.

## 4. Discussion

While some factors have already been identified for their correlation with prognosis, the prognostic factors for response to CAR-T-cell therapy are a matter of ongoing research [12,13,14]. Among them, a lower LDH level before lymphodepleting therapy preceding CAR-T-cell infusion was associated with superior progression-free survival [12,13], while the achievement of remission within the first three months after CAR-T-cell therapy was shown to be associated with a durable response [5,17,19]. Other parameters, e.g., whether distinct lymphoma subtypes respond better to CAR-T-cell therapy than others, currently remain unclear. In this study, performed at a single tertiary academic center, we focused on the prognostic significance of transformed/secondary versus de novo/primary DLBCL histopathology in a cohort of subsequent DLBCL patients undergoing CAR-T-cell treatment.

Strikingly, the patients with transformed lymphoma had a higher CR rate than the de novo patients (78% in the transformed group vs. 33% in the de novo group; *p* = 0.0176). Furthermore, patients with de novo lymphoma had a high relapse rate of 67% and a high mortality rate of 56% due to lymphoma progression in all cases. In contrast, the relapse rate was only 28%, and the mortality rate was 22% in the transformed lymphoma group. PFS and OS were significantly better for patients with transformed lymphoma as compared to de novo disease. The median PFS for de novo lymphoma was 3 months, and the median OS was 6.6 months, whereas the median PFS and OS were not reached in the transformed lymphoma patients (for PFS, *p* = 0.0093; for OS, *p* = 0.0209).

Achieving a CR within the first six months after CAR-T-cell infusion was associated with a more favorable outcome in our cohort, consistent with the previous literature [1,4,13]. A difference between the patients who did and did not achieve CR was statistically significant, both within the whole cohort (for PFS *p* < 0.0001 and for OS *p* < 0.0001), as well as within each of the histological groups (in the de novo group with *p* = 0.0239 for PFS and *p* = 0.0215 for OS; in the transformed group with *p* < 0.0001 for both PFS and OS). Other parameters, including the number of previous therapy lines, bridging therapy, LDH concentration at the time of the CAR-T-cell infusion, the manifestation of CRS and ICANS, peak CAR-T DNA concentration in the peripheral blood, and their dynamics had no significant impact on the outcomes following CAR-T-cell therapy. This, however, may be due to the limited size of our cohort.

Our data suggest that the histological subtype of a transformed/secondary DLBCL is predictive of a better response and outcome to CAR-T-cell therapy than de novo/primary DLBCL. To our knowledge, this aspect was not yet adequately reported. In a retrospective study, Chong et al. found sustained PFS in patients with follicular lymphoma (median PFS was 26.2 months, and 43% of all patients remained progression-free at 5 years) following CAR-T-cell therapy. On the other hand, a group of patients with DLBCL showed somewhat inferior outcomes (median PFS was 5.8 months and progression-free survival at 5 years was 31%) [16]. The TRANSCEND trial, a core clinical study assessing the benefit of a lisocaptagene maraleucel CAR-T treatment, reported a notable duration of response in patients with DLBCL transformed from follicular lymphoma and patients with primary mediastinal B-cell lymphoma [4].

Our results, in combination with the studies cited above, suggest that aggressive lymphoma derived from an indolent type (e.g., follicular lymphoma) may represent a group of lymphoma patients with a more favorable response to CAR-T-cell therapy. Genetic features, e.g., special intrinsic features of transformed DLBCL, could explain this difference, but the causes underlying our observation await to be elucidated. In fact, adequately powered prospective studies or larger real-world cohorts will be needed to confirm our results and to identify additional parameters responsible for these differences, e.g., primary and secondary genetic events that may be relevant. These approaches will be helpful to better identify those patients with DLBCL that could preferentially benefit from CAR-T-cell therapy and to stratify patients according to their individual relapse risk following CAR-T-cell therapy. Finally, these approaches may also be helpful to identify patients with a need for more frequent monitoring and, eventually, additional therapeutic intervention following CAR-T-cell therapy, whereas others may not need such intensified approaches. Some patients may benefit, however, from other novel adoptive immunotherapies (e.g., CAR-NK cells, γδ T cells, as well as non-conventional MR1-dependent TCRs and NKG2D CAR-T cells) instead of the conventional CAR-T-cells. Some of these novel approaches, which are the focus of intensive research, are promising for larger-scale manufacturing and universal application [20,21].

## 5. Conclusions

Our data suggest that transformed/secondary lymphoma is associated with a more favorable outcome after CAR-T-cell therapy compared to de novo/primary lymphoma. However, larger studies may be needed to address this question ultimately and, thereby, to identify those patients who would benefit the most from CAR-T-cell therapy.

## Figures and Tables

**Table 1:** Clinical characteristics of the patients.

Parameter	Result
Patients	36
Demographic characteristics	
Males:females (ratio)	20:16 (1.2)
Median age at the time of CAR-T-cell therapy (range)	68.5 (27–79)
Initial diagnosis	
DLCBL	36 (100%)
Primary (de novo) DLBCL	18 (50%)
Secondary/transformed DLBCL	18 (50%)
FL	13 (36%)
CLL/SLL	3 (8%)
MZL	1 (3%)
Other	1 (3%)
Initial lymphoma stage	36
I	1 (3%)
II	5 (14%)
III	6 (17%)
IV	17 (47%)
Unknown	7 (19%)
B-symptoms at lymphoma diagnosis	22 (61%)
Radiotherapy before lymphapheresis	12 (33%)
Radiotherapy as bridging between lymphapheresis and CAR-T-cell infusion	4 (11%)
Previous hematopoietic stem-cell transplantation before CAR-T-cell therapy	22 (61%)
Autologous SCT	21 (58%)
Allogeneic SCT	1 (3%)
IPI for lymphoma patients	36
1	0 (0%)
2	3 (8%)
3	4 (11%)
4	3 (8%)
5	1 (3%)
Unknown	25 (70%)
Number of treatment lines before CAR-T-cell therapy	
2	22 (61%)
3	9 (25%)
>3	5 (14%)

CAR-T: chimeric antigen receptor T cell; DLCBL: diffuse large B-cell lymphoma; FL: follicular lymphoma; CLL: chronic lymphocytic leukemia; SLL: small lymphocytic lymphoma; MZL: marginal-zone lymphoma; SCT: hematopoietic stem-cell transplantation; IPI: International Prognostic Index.

**Table 2:** Clinical status of the patients at the time of CAR-T-cell treatment.

Remission Status	Number (%)
CR	2 (6%)
PR	9 (25%)
SD	8 (22%)
PD	17 (47%)
Bridging therapy given: Yes	17 (47%)
Radiotherapy	4 (11%)
Pharmacotherapy	17 (47%)
MATRIx	1 (3%)
R-GEMOX	2 (6%)
Ibrutinib	4 (11%)
Gemcitabine	1 (3%)
Bendamustine	6 (17%)
Polatuzumab vedotin	4 (11%)
Rituximab	9 (25%)
CAR-T-cell therapy type	
Kymriah^®^ (Novartis)	26 (72%)
Yescarta^®^ (Gilead)	10 (28%)
Lymphodepleting chemotherapy	
Fludarabine, Cyclophosphamide	36 (100%)

CAR-T: chimeric antigen receptor T cell; CR: complete remission; PR: partial remission; SD: stable disease; PD: progressive disease; MATRIx: methotrexate, cytarabine, thiotepa, and rituximab; R-GEMOX: gemcitabine, oxaliplatin, and rituximab.

**Table 3:** Clinical course after CAR-T-cell treatment.

Parameter	Number (%)
Cytokine-release syndrome	24 (67%)
Grade 1	6 (17%)
Grade 2	15 (42%)
Grade 3	2 (6%)
Grade 4	1 (3%)
CAR-T-Related Encephalopathy Syndrome	14 (39%)
Grade 1	4 (11%)
Grade 2	4 (11%)
Grade 3	4 (11%)
Grade 4	2 (6%)
Median duration of hospitalization, days (range)	21.5 (18–51)

**Table 4:** Clinical course and characteristics of the patients after CAR-T-cell therapy. Comparison of the two groups of patients with transformed versus de novo lymphoma. The *p*-values were calculated using Chi-square tests.

Parameter	Result		
	Transformed *n* = 18	De Novo *n* = 18	*p*-Value
Best response after the CAR-T-cell therapy			
CR	14 (78%)	6 (33%)	0.0200
PR	2 (11%)	5 (28%)	
SD	1 (6%)	5 (28%)	
PD	1 (6%)	2 (11%)	
Best response achieved at different intervals from CAR-T-cell infusion, months			
1	4 (22%)	15 (83%)	0.0015
3	11 (61%)	2 (11%)	
6	3 (17%)	1 (6%)	
Final response at last follow-up			
CR	14 (78%)	5 (28%)	0.0057
PR	0 (0%)	2 (11%)	
SD	0 (0%)	0 (0%)	
PD	4 (22%)	11 (61%)	
Cytokine-release syndrome	11 (61%)	13 (72%)	
Grade 1	3 (17%)	3 (17%)	0.5274
Grade 2	7 (39%)	8 (44%)	
Grade 3	1 (6%)	1 (6%)	
Grade 4	0 (0%)	1 (6%)	
CAR-T-Related Encephalopathy Syndrome	8 (44%)	6 (33%)	
Grade 1	2 (11%)	2 (11%)	0.2308
Grade 2	4 (22%)	0 (0%)	
Grade 3	2 (11%)	2 (11%)	
Grade 4	0 (0%)	2 (11%)	
Relapse	5 (28%)	12 (67%)	
Mortality (all due to disease progression)	4 (22%)	10 (56%)	

CAR-T: chimeric antigen receptor T cell; CR: complete remission; PR: partial remission; SD: stable disease; PD: progressive disease.

## Data Availability

The data presented in this study are available in this article.
